# Immunization With a Secreted Esterase Protects Mice Against Multiple Serotypes (M1, M3, and M28) of Group A *Streptococcus*

**DOI:** 10.3389/fmicb.2020.00565

**Published:** 2020-04-03

**Authors:** Xiaolan Zhang, Deqin Wei, Yuan Zhao, Zhaohua Zhong, Yue Wang, Yingli Song, Minghui Cai, Wenli Zhang, Jizi Zhao, Chunmei Lv, Hui Zhu

**Affiliations:** College of Basic Medical Sciences, Harbin Medical University, Harbin, China

**Keywords:** group A *streptococcus* (GAS), protective effect, streptococcal secreted esterase (Sse), immunization, mice

## Abstract

Streptococcal secreted esterase (Sse) is a platelet-activating factor acetylhydrolase that is critical for Group A *Streptococcus* (GAS) skin invasion and innate immune evasion. There are two Sse variant complexes that share >98% identity within each complex but display about 37% variation between the complexes in amino acid sequences. Sse immunization protects mice against lethal infection and skin invasion in subcutaneous infection with the hypervirulent CovRS mutant strain, MGAS5005. However, it is not known whether Sse immunization provides significant protection against infection of GAS with functional CovRS and whether immunization with Sse of one variant complex provides protection against infection of GAS that produces Sse of another variant complex. This study was designed to address these questions. Mice were immunized with recombinant Sse of M1 GAS (Sse^M1^) and challenged with MGAS5005 (serotype M1, CovS mutant, and Sse of variant complex I), MGAS315 (M3, CovS mutant, and Sse of variant complex I), MGAS2221 (M1, wild-type CovRS, and Sse of variant complex I), and MGAS6180 (M28, wild-type CovRS, and Sse of variant complex II). Sse^M1^ immunization significantly increased survival rates of mice in subcutaneous MGAS5005 and intraperitoneal MGAS6180 challenges and showed consistently higher or longer survival in the other challenges. Immunized mice had smaller skin lesion and higher neutrophil responses in subcutaneous infections and lower GAS burdens in spleen, liver, and kidney in most of the challenge experiments than control mice. Sse^M1^ immunization enhanced proinflammatory responses. These data suggest that Sse immunization has a broad benefit against GAS infections that can vary in extent from strain to strain and that the benefit may be due to the immunization-enhanced proinflammatory responses. In particular, immunization with Sse^M1^ can provide protection against M28 GAS infection even though its Sse and Sse^M1^ have significant variations.

## Introduction

*Streptococcus pyogenes* also called Group A *Streptococcus* (GAS), can produce an arsenal of extracellular secreted proteins to evade the innate immune system (Walker et al., 2014; Hynes and Sloan, 2016; Liu and Lei, 2018; Happonen et al., 2019). Although treated with modern medicine, it remains a significant cause of global morbidity and mortality (Dickson et al., 2018; Hua et al., 2019; Lynskey et al., 2019). Historically, many of these secreted proteins have been studied to find the mechanisms by which they facilitate the resistance against host defenses as well as to find possible candidate antigens for vaccines. For example, the extracellular peptidases ScpA and SpyCEP reduce neutrophil recruitment by degrading the chemotactic C5a peptide and IL-8/CXC chemokines. Streptolysins S (SLS) and O (SLO) are pore-forming toxins that can damage immune cells (Flaherty et al., 2015; Uchiyama et al., 2015). Sse is a carboxylic acid esterase similar to that found in humans and hydrolyzes platelet-activating factor (PAF) (Liu et al., 2012, 2013). PAF serves as a phospholipid mediator manufactured by endothelial cells, neutrophils, macrophages, and granular eosinophils (Chaithra et al., 2018). PAF mediates IL-1β-induced chemotaxis of natural killer cells and neutrophilsand can induce the migration of neutrophils to infection sites (Liu et al., 2019). Liu et al. reported the protective effect of Sse immunization against skin infection with M1 strain MGAS5005 (Liu et al., 2007), a hypervirulent CovS mutant. CovS is the sensor of CovRS (also known as CsrRS), the two-component regulatory system of virulence, that negatively regulates many virulence factors (Heath et al., 1999). CovS mutation enhances expression of many virulence factors including Sse (Sumby et al., 2006; Li et al., 2013). In addition, there are two Sse variant complexes among GAS isolates (Liu et al., 2007). Complex I includes Sse from strains M1, M2, M3, M5, M6, M12, and M18, and complex II includes the Sse proteins that are produced by M4, M28, and M49. The proteins within each complex share >98% identity in amino acid sequence and there is 63% sequence identity between the complexes. These observations raise two important questions regarding the immunogenicity of Sse. First, does Sse immunization provide significant protection against infection of GAS with functional CovRS? Second, does immunization with Sse of one Sse variant complex provide protection against infection of GAS that produces Sse of another variant complex? To address these questions, we prepared recombinant Sse protein derived from the M1 strain MGAS5005, Sse^M1^, and characterized its capacity to provide protective immunity against MGAS2221, MGAS315, and MGAS6180 infection. Sse^M1^ immunization protects mice against MGAS5005 and MGAS315 in subcutaneous infection route, while it protects mice against MGAS5005, MGAS315, MGAS2221, and MGAS6180 in intraperitoneal infection route.

## Materials and Methods

### Bacterial Strains and Culture Conditions

Hypervirulent Serotype M1 CovRS mutant strain MGAS5005 (Li et al., 2013), wild-type M1 strain MGAS2221 (Sumby et al., 2006), hypervirulent M3 CovRS mutant strain MGAS315 (Stetzner et al., 2015), and wild-type M28 strain MGAS6180 (Stetzner et al., 2015) were grown in Todd-Hewitt broth (Becton, Dickinson Company) supplemented with 0.2% yeast extract (THY) at 37°C with 5% CO_2_.

### Gene Cloning, Protein Expression and Purification

The *sse*-MGAS5005 gene was cloned into the pET21b-his vector (Novagen, Madison, WI, United States) to produce the recombinant plasmid (pET21b-his-*sse*), and the recombinant plasmid was transformed into *E. coli* BL21 (DE3) to induce the expression of Sse^M1^ protein. The Sse^M1^ protein was purified by chromatography using DEAE ion-exchange chromatography and nickel resin affinity chromatography, as previously described (Liu et al., 2007). Protein was concentrated using a Centricon Plus 20 filtration device (Millipore, Bedford, MA, United States), and the protein concentration was determined by a BCA protein assay kit.

### Active Immunization and Anti-Sse IgG Detection

Female CD1 mice (4 to 5 weeks old) were purchased from Department of Experimental Animals of Harbin Medical University and used in the immunization study. The purified Sse^M1^ protein (dissolved in 20 mM Tris–HCl, pH 8.0) was mixed with aluminum-containing adjuvant (ALUM). Two groups of mice were subcutaneously immunized three times at days 0, 14, and 28 with ALUM or Sse^M1^ (50 μg). Blood samples were collected at days 0, 14, 28, and 42 via tail bleeding, and then serum was separated and stored at −80°C for anti-Sse IgG detection.

The mouse IgG ELISA kit (Neo Bioscience) was used to measure serum IgG concentration. The standard was added to the blank wells, while 100 μl different concentration standard or samples were added to other wells. Then the plate was incubated for 1.5 h at 37°C. The plate was washed five times, and then biotinylated antibody dilutions were added to the blank wells while 100 μl enzyme labeled antibody dilutions were added to other wells. The plate was incubated for 1 h at 37°C. The plate was washed five times again, and then dilutions were added to the blank wells while 100 μl enzyme conjugated dilutions were added to other wells. The plate was incubated in a dark place for 30 min at 37°C. The plate was washed five times and then 100 μl 3,3′,5,5′-tetramethylbenzidine (TMB) was added to each well for 15 min at 37°C away from light. The colorimetric reaction was stopped by the addition of 100 μl ending solution. To calculate IgG concentration, A_450_ was measured using a microplate reader. The IgG concentration of each sample was given by the formula from the standard curve: A_450(sample)_-A_450(blank)_ = 2.534x + 0.157 (x: mg/ml, *R*^2^ = 0.97).

### Mouse Infection and Immunization Experiments

The protocols for the infection and immunization described below were approved by the Institutional Research Board of Harbin Medical University. Mice were anesthetized with inhalation of isoflurane prior to all procedures.

As described above, female CD1 mice were subcutaneously injected with 200 μl of mixture containing 50 μg Sse^M1^ and 50 μl ALUM or ALUM only on day 0 and boosted with the same treatment on days 14 and 28. At 2 weeks after the second booster, Sse^M1^-immunized and control mice were infected subcutaneously or intraperioteally with MGAS5005, MGAS2221, MGAS315, or MGAS6180, as previously described (Stetzner et al., 2015; Zhang et al., 2017). GAS was harvested at the exponential growth phase, washed with pyrogen-free phosphate-buffered saline (PBS), and resuspended in PBS.

In subcutaneous infection model (SC), Sse^M1^-immunized and adjuvant control mice were subcutaneously inoculated with 0.2 ml (∼1.0 × 10^8^CFU) of a bacterial suspension for each strain. Mice (9–10 mice/group, total eight groups) from each group were monitored daily for 15 days to determine survival rates. The remaining mice of each group were euthanized at 24 h after inoculation for the measurements of skin lesion sizes (5 mice/group), GAS burdens in spleen, liver, and kidney (6–8 mice/group), myeloperoxidase (MPO) activity (5 mice/group) at infection sites, and cytokines levels (5–6 mice/group).

In intraperitoneal infection model (IP), Sse-immunized and adjuvant control mice were intraperitoneally injected with 0.2 ml (∼1.0 × 10^8^CFU) of GAS bacterial suspension for each strain. Mice (9–10 mice/group, total eight groups) from each group were monitored to determine survival rates. The other mice (7–8 mice/group) of each group were euthanized at 24 h after inoculation or within 24 h if they were moribund to collect the spleen, liver, and kidney for measuring GAS burdens.

### Measurement of GAS Burdens at Skin Infection Sites and Organs

The liver, spleen, and kidneys were collected and weighed. The organs were homogenized in PBS using Kontes pestles. The homogenates were serially diluted in PBS and plated on THY agar plates for the quantification of bacterial CFUs. The CFU count was expressed as Log_10_ CFU/100 mg tissue.

### Measurement of Skin Lesion Size

The skin around the infection site was peeled off, and the lesions were imaged with a ruler. Areas of lesions were measured by analyzing the images using the area measurement tool of Adobe Acrobat 9 software.

### The Myeloperoxidase (MPO) Activity

Twenty-four hours post SC infection, the skin lesions of mice immunized with ALUM or SsE^M1^ were excised and homogenized. The level of MPO activity (U/total) in the homogenate was determined as the absorbance value at 450 nm wavelength by a microplate reader (SpectraMax) using an MPO assay kit (Nanjing Jiancheng Bioengineering Institute) that is based on the previously reported MPO assay (Stetzner et al., 2015).

### Cytokine Detection by ELISA

Twenty-four hours post SC infection, the skin lesions were excised and homogenized, as described above. The homogenates were centrifuged to obtain supernatant. The levels of TNF-α, IL-2, IL-1β, and IFN-γ concentrations inthe supernatants were quantified using enzyme-linked immunosorbent assay (ELISA) according to the manufacturer’s instructions (Elabscience Biotechnology Co., Ltd.).

### Statistical Analysis

The survival data were analyzed by the log-rank test. IgG concentrations were analyzed by two-way ANOVA with Bonferroni multiple comparison post-test. The other data were analyzed by unpaired t-test. GraphPad Prism 6 software was used for these statistical analyses. The data were considered to be statistically significant if *P* values < 0.05.

## Results

### Sse^M1^ Elicits a Robust IgG Response After Immunization

**FIGURE 1:**
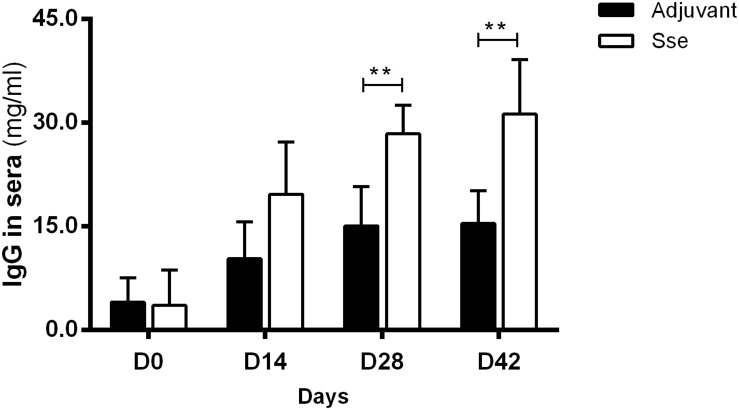
The IgG response in the sera after subcutaneous immunization. Mice were administered recombinant Sse^M1^ or adjuvant alone and the resulting serum IgG concentrations were determined by ELISA. Symbols in statistical analyses: **P < 0.01.

To examine the antigenic potential of Sse^M1^, recombinant Sse^M1^ and adjuvant ALUM was administered subcutaneously to mice three times at days 0, 14, and 28. The anti-Sse^M1^IgG concentrationin sera of Sse-immunized mice were evaluated and compared with those of control mice with ALUM treatment. As shown in Figure 1, mice immunized with Sse^M1^ developed a significantly increased anti-Sse^M1^ IgG response that became almost saturated at day 28. The IgG concentration at day 42 in Sse^M1^-immunized mouse serum was 2-fold higher than that in adjuvant only-immunized mouse serum (*P* < 0.01). Thus, in subcutaneous immunization of mice, Sse^M1^ is an effective antigen which elicts a robust IgG response.

### Variation in Protective Effect of Sse^M1^ Immunization in Mice Against Subcutaneous Infections With MGAS5005, MGAS2221, MGAS315, and MGAS6180

**FIGURE 2:**
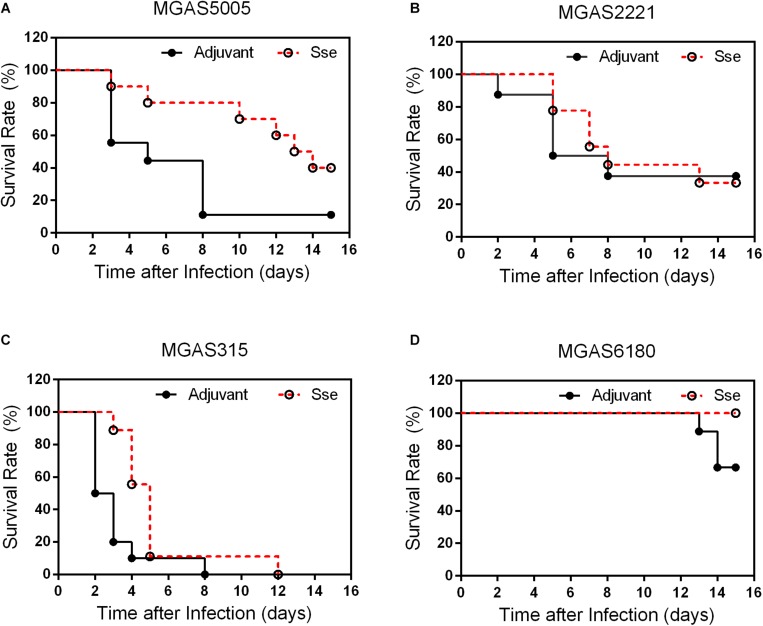
Effects of Sse^M1^ Immunization on survival of mice subcutaneous MGAS5005, MGAS315, MGAS2221 and MGAS6180 infections. Sse^M1^-immunized and adjuvant control mice were subcutaneously inoculated via the dorsal surface with 1.0 × 10^8^CFU of each strain. Presented are the survival rates of mice in infections with MGAS5005 **(A)**, MGAS2221 **(B)**, MGAS315 **(C)**, and MGAS6180 **(D)**. *P* values in the figure were from the Log-rank test. (MGAS5005: *P* = 0.0298; MGAS2221: *P* = 0.8428; MGAS315: *P* = 0.0133; MGAS6180: *P* = 0.0657).

To determine whether Sse^M1^ immunization provides significant protection against infection of GAS with functional CovRS (Question 1) and whether immunization with Sse^M1^ of Sse variant complex I provides protection against infection of GAS that produces Sse of Sse variant complex II (Question II), Sse^M1^-immunized and control mice were infected subcutaneously with MGAS5005, MGAS2221, MGAS315 and MGAS6180. At day 15 after inoculation, 40% of immunized mice with MGAS5005 infection survived whereas the survival rate of the control mice was 11% (Log-Rank test: *P* = 0.0298) (Figure 2A), and the median survival time of Sse^M1^-immunized mice after infection with MGAS5005 was 2-fold higher than that of control mice, 13.5 days compared to 5.0 days. Thus, Sse^M1^ immunization significantly protects mice against subcutaneous MGAS5005 infection, confirming the previous finding (Liu et al., 2007). MGAS5005 is hypervirulent due to a CovS non-sense mutation that lead to high expression of many virulence factors including Sse (Li et al., 2013). MGAS315 is a hypervirulent M3 CovS missense mutant (Stetzner et al., 2015). Sse^M1^ and Sse produced by MGAS315 share 99.7% identity with a single amino acid residue variation. The survival curves of Sse^M1^-immunized and control mice in subcutaneous MGAS315 infection are significantly different (Log-rank test: *P* = 0.0133) (Figure 2C). It should be noted that all Sse^M1^-immunized mice in MGAS315 infection were moribund (Figure 2C), which could be due to the higher virulence of M3 GAS (Stetzner et al., 2015). Sse^M1^ immunization did not lead to significant difference in survival curves in the infection of the immunized and control mice with wild-type M1 strain MGAS2221 (Figure 2B).

These results indicate that Sse^M1^ immunization has higher protective potential against hypervirulent CovRS mutants as evaluated on virulence. As for question II, 3 of 10 control mice were moribund whereas all Sse^M1^-immunized mice survived subcutaneous infection with MGAS6180 (Log-rank test: *P* = 0.0657) (Figure 2D). The virulence data suggest that Sse^M1^ immunization has a protective effect against MGAS6180 infection but the protection is not statistically significant in this virulence test. MGAS6180 is not highly virulent in subcutaneous infection of mice, making it more difficult to evaluate the protective effect of Sse^M1^ immunization against MGAS6180. Increasing numbers of mice or GAS dose would be one way to convincingly address question II using the subcutaneous infection model. We chose to use the intraperitoneal infection model to address question II that is described later.

**FIGURE 3:**
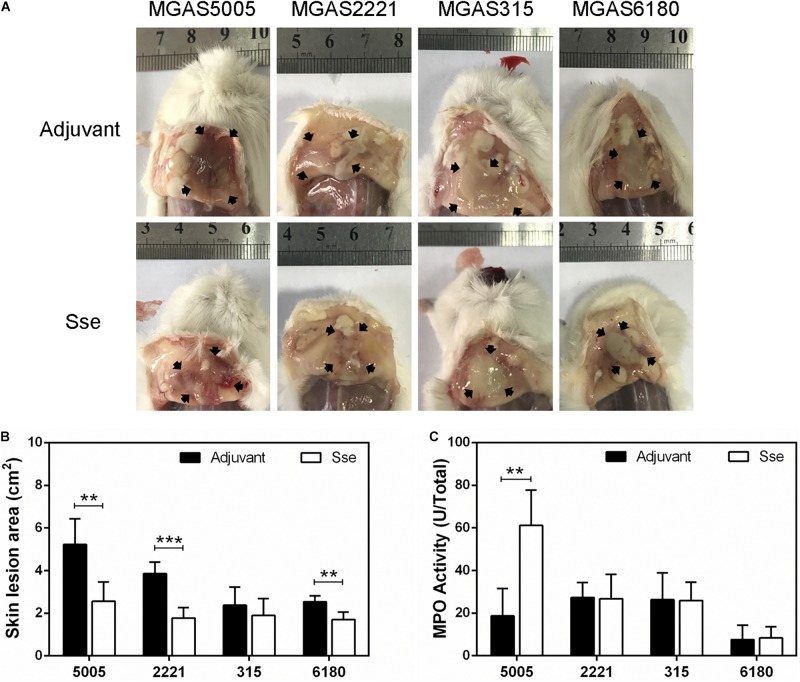
Effect of Sse^M1^ immunization on skin invasion in subcutaneous GAS infection. Sse^M1^-immunized and adjuvant control mice were subcutaneously inoculated with 1.0 × 10^8^ CFU of each strain and euthanized at 24 h post inoculation for measuring lesion size and MPO activities. **(A)** Representative images of skin lesions in mice infected with MGAS5005, MGAS2221, MGAS315 and MGAS6180. The abscess was indicated by the black arrows. **(B)** The skin lesion area in Sse^M1^-immunized (open bars) and adjuvant control (solid bars) mice. **(C)** Total MPO activity in the skin infection site in Sse^M1^-immunized (open bars) and adjuvant control (solid bars) mice. Symbols in statistical analyses: **P < 0.01 and ***P < 0.001.

The protective effects of Sse^M1^ immunization against GAS infections were further examined by comparing skin invasion and systemic dissemination in the subcutaneous infections. Figure 3A shows representative inside-out images of skin infection sites from Sse^M1^-immunized and control mice infected with the four strains. The mean skin lesion area of the immunized mice in MGAS5005 infection was smaller than those of the control mice (*P* = 0.0036) (Figure 3B). It is interesting that Sse^M1^ immunization significantly reduced the lesion size in MGAS2221 and MGAS6180 infections in comparison with the control mice (MGAS2221, *P* = 0.0002; MGAS6180, *P* = 0.0024). Thus, Sse^M1^ immunization protects mice against skin invasion by wild-type M1 and M28 GAS. In contrast, Sse^M1^ immunization did not affect skin invasion significantly in MGAS315 (*P* = 0.3843).

**FIGURE 4:**
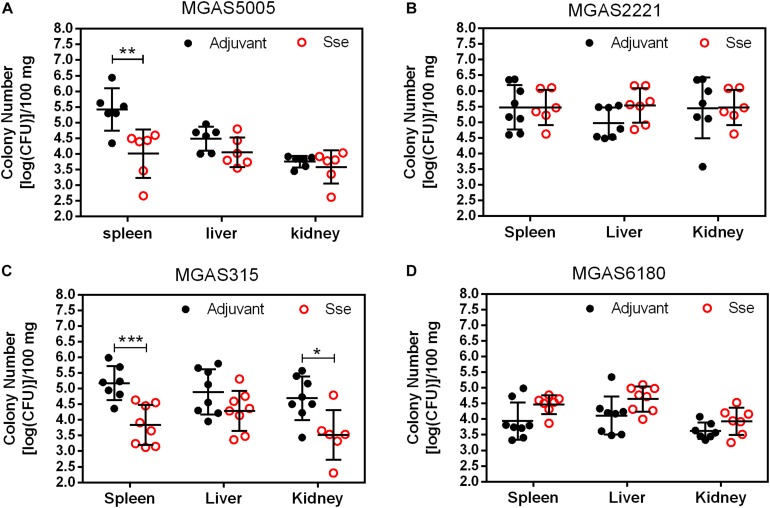
GAS burdens in the spleen, liver, and kidney of mice at 24 h post subcutaneous inoculation of MGAS5005 **(A)**, MGAS2221 **(B)**, MGAS315 **(C)**, and MGAS6180 **(D)**. Organ homogenates were serially diluted in PBS and plated on THY agar plates for the quantification of bacterial CFUs. Symbols in statistical analyses: *P < 0.05; **P < 0.01; and ***P < 0.001.

As shown in Figure 4, GAS burdens in spleen and kidney in SsE^M1^-immunized mice were significantly lower than those in control mice in subcutaneous MGAS5005 and MGAS315 infections whereas Sse^M1^ immunization did not alter GAS burdens in these organs in MGAS2221 and MGAS6180 infections. These GAS burden data appeared to be correlated with the virulence data in Figure 2. These results suggest that Sse^M1^immunization can affect skin invasion and/or systemic infection depending on strains.

### Immunization With Sse^M1^ Protects Mice Against Intraperitoneal GAS Infection

**FIGURE 5:**
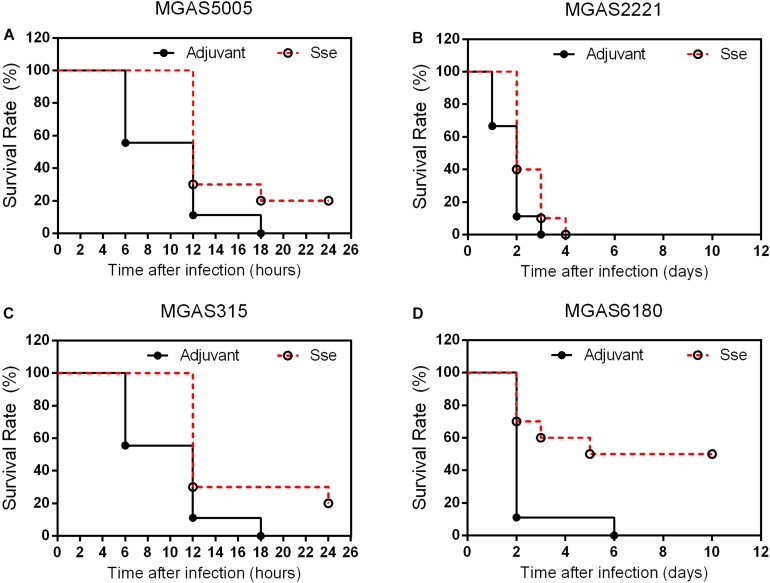
Effects of Sse^M1^ immunization on survival of mice in intraperitoneal MGAS5005, MGAS2221, MGAS315, and MGAS6180 infections. Sse^M1^-immunized and adjuvant control mice were intraperitoneally inoculated with 1.0 × 10^8^CFU of each strain. Presented are the survival rates of mice in infections with MGAS5005 **(A)**, MGAS2221 **(B)**, MGAS315 **(C)**, and MGAS6180 **(D)**. *P* values in the figure were from the Log-rank test. (MGAS5005:*P* = 0.0276; MGAS2221:*P* = 0.0409; MGAS315:*P* = 0.0150; MGAS6180:*P* = 0.0078).

**FIGURE 6:**
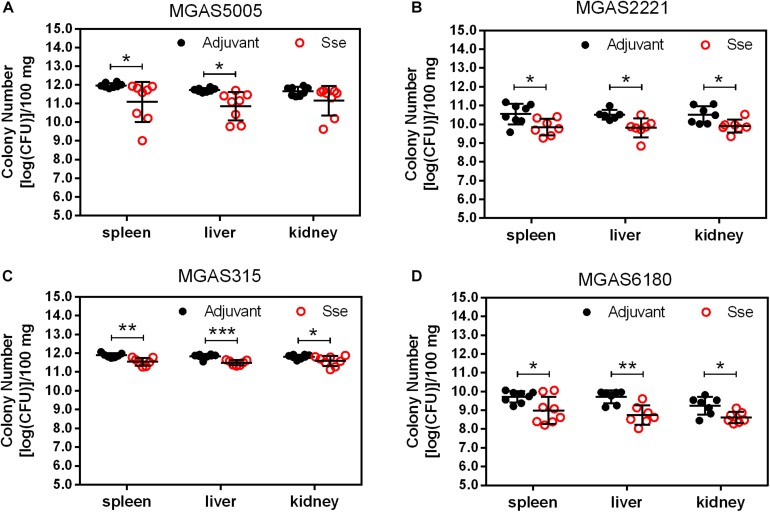
GAS burdens in the spleen, liver, and kidney of mice at 24 h post intraperitoneal inoculation of MGAS5005 **(A)**, MGAS2221 **(B)**, MGAS315 **(C)**, and MGAS6180 **(D)**. Organ homogenates were serially diluted in PBS and plated on THY agar plates for the quantification of bacterial CFUs. Symbols in statistical analyses: *P < 0.05, **P < 0.01, and ***P < 0.001.

**FIGURE 7:**
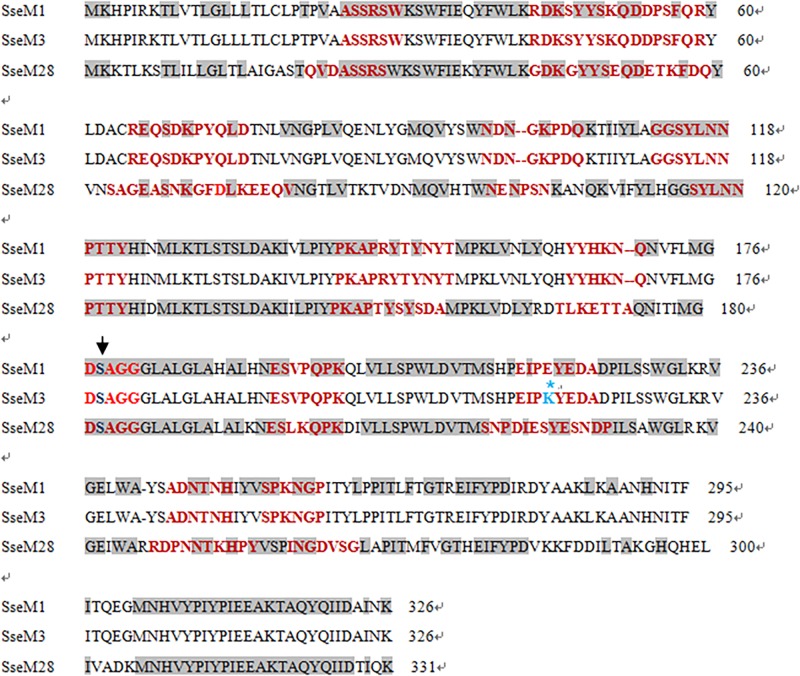
Amino acidsequenceand epitope alignment of Sse^M1^, Sse^M3^, and Sse^M28^. The conserved catalytic serine residue is indicated with the arrow. Gray boxes refer to consensus sequences and red texts refer to the predicting epitopes. Sequences between SsE^M1^ and SsE^M3^ are identical with the exception of the No. 220 residue (E→K). Sse sequences: Sse^M1^ (GenBank: AAZ52025.1, from MGAS5005), Sse^M3^ (GenBank: AAM80100.1, from MGAS315), Sse^M28^ (GenBank: AAX72560.1, from MGAS6180).

Since the subcutaneous infection model was insufficient to evaluate the protective effect of Sse^M1^ immunization against MGAS6180 infection because of its low virulence in subcutaneous infection, we examined the protection of Sse^M1^ immunization against intraperitoneal infection with MGAS6180 and the three other strains. Even though the majority of Sse^M1^-immunized mice were moribund in intraperitoneal MGAS5005, MGAS2221, and MGAS315 infections, these immunized mice survived significantly longer than control mice (Log-rank test: *P* < 0.05) (Figures 5A–C). Immunization with Sse^M1^ provided the stronger protection against MGAS6180 than against the other strains. All control mice infected with MGAS6180 were moribund whereas 50% of Sse^M1^-immunized mice survived in MGAS6180 infection (Log-rank test: *P* < 0.01) (Figure 5D). Sse^M1^ immunization significantly reduced GAS burdens in the spleen, liver, and kidney at 24 h after intraperitoneal inoculation (Figure 6), consistent with the survival data in Figure 5. Sse^M1^ immunization provides strong protection against intraperitoneal MGAS6180 infection even though its Sse has 37% variation in amino acid sequence in comparison with Sse^M1^ (Figure 7).

### Sse^M1^ Immunization Enhances Inflammatory Response at Skin Infection Model

It is known that Sse contributes to inhibition of neutrophil infiltration (Liu et al., 2012, 2013). Thus, we measured the total MPO activity at skin GAS infection sites in immunized and control mice. Among MGAS5005-infected mice, the total MPO activity at MGAS5005 infection sites of Sse^M1^-immunized mice (61.18 ± 6.75 U) was 3.3-fold higher than that of control mice (18.67 ± 5.73 U) (*P* = 0.0012) (Figure 3C). We did not detect any changes in the total MPO activity at MGAS2221, MGAS315 or MGAS6180 sites between immunized and control mice. A potential reason for these results might be that we measured the total MPO activity that did not account for the reduction of lesion size in Sse^M1^-immunized mice.

**FIGURE 8:**
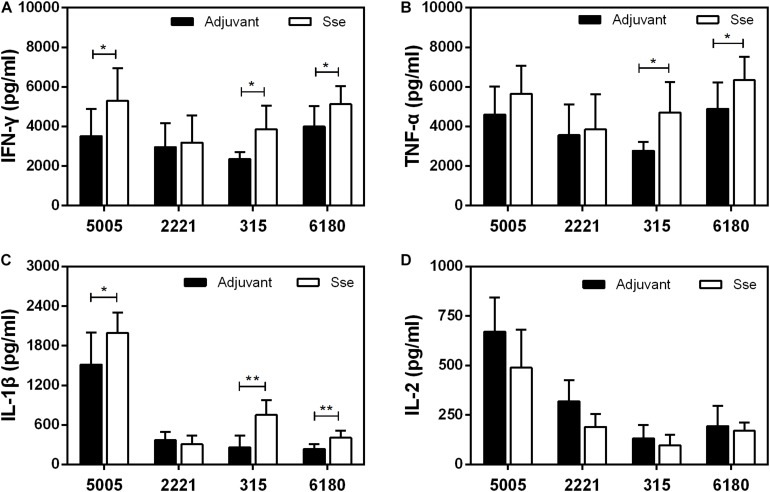
Effect of Sse^M1^ immunization on cytokine response at subcutaneous infection sites. Sse^M1^-immunized and adjuvant control mice were subcutaneously infected with 1.0 × 10^8^ CFU of MGAS5005, MGAS2221, MGAS315, and MGAS6180, and skin infection sites were collected at 24 h after inoculation for cytokine measurement. Presented are levels of IFN-γ **(A)**, TNF-α **(B)**, IL-1β **(C)**, and IL-2 **(D)** in the infected skin. Symbols in statistical analyses: *P < 0.05 and **P < 0.01.

We also compared levels of inflammatory cytokines at skin infection sites at 24 h after inoculation. The levels of IFN-γ, TNF-α and IL-1β, but not IL-2, at MGAS5005, MGAS315, and MGAS6180 infection sites were higher in Sse^M1^-immunized mice than in control mice (Figure 8). Since Sse hydrolyzes PAF, and PAF plays an important role in inflammation, Sse ^M1^ immunization may inhibit Sse-mediated PAF hydrolysis, thus leading to enhanced inflammation and protection.

## Discussion

Group A *Streptococcus* esterases represent targets for molecular therapies. The streptococcal esterase Sse hydrolyzes platelet-activating factor to impede neutrophil recruitment and facilitate innate immune evasion (Liu et al., 2007, 2012, 2013, 2015,Zhu et al., 2009). There are two variant complexes: I and II. Complex I includes M1, M2, M3, M5, M6, M12, and M18, whereas complex II includes M4, M28, and M49. In this study, we purified recombinant Sse^M1^ from MGAS5005 (*emm*1) and determined whether immunization with Sse^M1^ could provide protection against infection with other GAS strains.

Sse^M1^ immunization induced a high level of antibody production, which was almost saturated at day 28. This finding suggests that Sse^M1^ is immunogenic and that only one additional booster is needed to increase the anti-Sse^M1^ IgG amount. Here, we adopted two infection models. First, Sse^M1^ immunized mice were subcutaneously infected with MGAS5005, MGAS2221, MGAS315, and MGAS6180 on day 42. Then we observed the 15-day survival, and found that Sse^M1^-immunized mice infected with MGAS5005 and MGAS315 showed protective effect compared with control mice, but there was no significant change in the survival rates of mice infected with MGAS2221 and MGAS6180. Furthermore, we checked the bacterial CFUs to examine dissemination from the initial site of inoculation to the organs. Additionally, Sse^M1^-immunized mice subcutaneously infected with MGAS5005 and MGAS315 had a fewer bacteria than control mice, but this result was not replicated in mice infected withMGAS2221 and MGAS6180. These results are consistent with the survival rate post subcutaneous infection results. Furthermore, we adopted another infection model-intraperitoneal inoculation with bacteria to study the Sse^M1^-mediated protection. In this model, the mortality of mice was much higher than that of the subcutaneously infected mice. Regardless of whether the mice were infected with the hypervirulent or hypovirulent strains, the mice died quickly, however, there was still a difference between Sse^M1^-immunized and control mice. These results showed that Sse^M1^ immunization protects mice against MGAS5005 and MGAS315 in subcutaneous infection route, while it protects mice against MGAS5005, MGAS315, MGAS2221, and MGAS6180 in intraperitoneal infection route.

The average skin lesion sizes were reduced in Sse^M1^ immunized mice as compared to control mice during infection with the *emm*1 strains MGAS5005 and MGAS2221. These results suggest immunization with Sse^M1^ dampens the severe inflammatory response in the skin and hinders the spread of bacteria to the organs, during infection with strains also having complex I Sse. Neutrophils are inflammatory cells that can reach the infection site and react quickly. Thus, the evasion of neutrophils is crucial for the survival and dissemination of GAS during infection (Fieber and Kovarik, 2014). We detected the release of MPO in the skin lesion, which is representative of the activity of neutrophils. Sse^M1^ immunization increased the release of MPO in response to infection with MGAS5005but had no such effect on the response to infection with other strains. We assume that Sse immunization would protect against infection with other serotypes that produce Sse of the same complex.

It remains incompletely understood how GAS escapes from the innate immune system. Macrophages and dendritic cells (DCs) are commonly viewed as central coordinators of immune responses and are both essential for the control of GAS infection, in part through the secretion of multiple proinflammatory cytokines (Fieber and Kovarik, 2014; Tsatsaronis et al., 2014). After infection with MGAS5005, MGAS315, or MGAS6180, the levels of three such cytokines (IFN-γ, TNF-α, and IL-1β), which are important for effective innate immunity against GAS, increased in Sse^M1^-immunized mice compared with control mice. IFN-γ is responsible for a variety of immune functions, including the M1 polarization of macrophages (Lemire et al., 2017; Westman et al., 2018; Matsumura et al., 2019). Classically activated macrophages participate in key proinflammatory responses and enhance antigen presentation and pathogen clearance (Imai et al., 2018; Valderrama and Nizet, 2018; Hafner et al., 2019). IL-1β is a neutrophil recruitment factor produced by macrophages and DCs upon exposure to GAS (Valderrama et al., 2017; Flaherty et al., 2018; Liu et al., 2019). Recent research has discovered that an optimal level of IL-1β must be produced to eliminate pathogens and avoid excessive tissue lesion formation and mortality (Castiglia et al., 2016; Flaherty et al., 2018). Thus, the survival of control mice producing inadequate amounts of these cytokines reduced because of impaired bacterial clearance. The IL-2 level was reduced in Sse^M1^-immunized mice compared to control mice. Activated T cells can release IL-2, which promotes T cell activation in adaptive immune responses (Zhu et al., 2009; Song et al., 2018). However, according to current knowledge, adaptive immune responses rarely participate in GAS infection. The combined functions of IL-2 and other cytokines in the immune responses against GAS are currently not known. However, these cytokines are involved in the clearance of GAS and determine the extent of the subsequent inflammatory response and the likely outcome of infection.

The sequence of selected Sse homologs is presented in Figure 7. Sse^M1^ of MGAS5005 and MGAS2221 shares >99% amino acid sequence identity with Sse^M3^ of MGAS315 and approximately 63% identity with Sse^M28^ of MGAS6180. We used four kinds of methods to analyze antigenic determinants: Parker hydrophilicity prediction, Chou-Fasmanbeta-turn prediction, Karplus and Schulz flexibility prediction and Emini surface accessibility prediction. The results indicated that there are 12 epitopes in Sse^M1^, Sse^M3^ or Sse^M28^. Most of these epitopes shared similar amino acid sequences, especially the 8^th^ epitope (DSAGG), in which the catalytic residue Ser^178^ is the core site of enzymatic activity. In the future, we will synthesize the amino acid segments of these epitopes to study their specific functions.

## Data Availability Statement

All datasets generated for this study are included in the article/supplementary material.

## Ethics Statement

The animal study was reviewed and approved by The Institutional Research Board of Harbin Medical University (HMUIRB20190015).

## Author Contributions

HZ and CL conceived the study. XZ wrote the first draft of the manuscript. HZ revised the manuscript. XZ, DW, and YZ participated in the whole research and collected data. YW, YS, and MC participated in part of the research. ZZ, WZ, and JZ gave good advice to the study.

## Conflict of Interest

The authors declare that the research was conducted in the absence of any commercial or financial relationships that could be construed as a potential conflict of interest.
